# Glycaemic control using mobile-based intervention in patients with diabetes undergoing coronary artery bypass—study protocol for a randomized controlled trial

**DOI:** 10.1186/s13063-023-07580-x

**Published:** 2023-09-13

**Authors:** Yangwu Song, Yifeng Nan, Wei Feng

**Affiliations:** Department of Cardiovascular Surgery, National Clinical Research Center of Cardiovascular Diseases, National Center for Cardiovascular Diseases, Fuwai Hospital, Chinese Academy of Medical Sciences and Peking Union Medical College, Beijing, People’s Republic of China

**Keywords:** Glycaemic control, Secondary prevention, Diabetes mellitus, Coronary artery bypass

## Abstract

**Background:**

Applying technology through the use of the Internet and mobile phones can help provide education and trained peer support for patients with diabetes after coronary artery bypass (CABG). We are conducting a randomized controlled trial to evaluate the efficacy and feasibility of mobile-based coaching intervention in improving risk-factor control and secondary prevention in patients with diabetes after CABG.

**Methods:**

The glycaemic control using miniprogram-based intervention in patients with diabetes undergoing coronary artery bypass to promote self-management (GUIDE ME) study is a multi-centre, randomized controlled trial of mobile intervention versus standard treatment with 6 months follow-up conducted in 2 hospitals in China. The interventions are education and a reminder system based on the WeChat mini-program. Participants in the intervention groups receive 180 videos (including lines) about secondary prevention education for 6 months as well as the standard treatment. Behavioural change techniques, such as prompting barrier identification, motivational skills, and goal setting, are employed. A total sample size of 820 patients would be adequate for the GUIDE ME study. The primary outcome is the change of glycaemic haemoglobin (HbA_1c_) at 6 months. Secondary outcomes include a change in the proportions of patients achieving HbA_1c_, fasting blood glucose, systolic blood pressure, low-density lipoprotein cholesterol (LDL-C) and medication adherence.

**Discussion:**

This trial is the first to investigate the efficacy of mobile phone WeChat-based video coaching and medication reminder mini-program system to improve self-management in patients with diabetes and coronary heart disease (CHD) after CABG and has the potential to be applied in resource-limited settings across diverse populations. If successful, such mobile intervention could be used and scaled up to improve care for this high-risk group of patients.

**Trial registration:**

ClinicalTrials, NCT04192409. Registered on December 10, 2019.

**Supplementary Information:**

The online version contains supplementary material available at 10.1186/s13063-023-07580-x.

## Background

Diabetes mellitus (DM) is associated with increased mortality and morbidity in patients undergoing coronary artery bypass grafting (CABG) specifically [1]. It is recommended that secondary prevention including long-term glucose control should ideally be optimized for this high-risk group of patients [1]. In low- and middle-income countries (LIMCs), including China, only one-third of individuals nationwide have adequate glycaemic control and over two-thirds of patients with coronary artery disease (CHD) take no medication [2, 3]. CABG patients with DM should receive coordinated medical care from a diabetes mellitus monitoring team. However, it is difficult to generalize and implement such interventions of patient-centred care given the limited access to education, consultation, and high costs of organization [4]. Given the high-risk profile of this subgroup among CHD patients, cost-effective and scalable interventions to enhance secondary prevention are urgently needed.

Applying technology through the use of the Internet and mobile phones can help provide education and trained peer support for patients after CABG, even for those who are unable to access cardiac rehabilitation because of geographic barriers [1]. As of August 2016, China had the largest number of mobile phone owners in the world. WeChat had become the most popular messaging communication app in China, which had a monthly-active-user of 549 million [5]. As such, WeChat has the potential to be a scalable and powerful tool to deliver health information.

There are thousands of mobile applications for supporting diabetes mellitus self-management, serving primarily as tracking and reference apps. In fact, < 1% of mobile applications have been evaluated through research and even fewer have demonstrated outcomes [6]. Prior studies showed inconsistent results. Evidence indicated that most trials to date have been designed to target a single condition and have not been based on behavioural change techniques (BCTs). Most of the studies were limited to single-centre studies and were underpowered [6, 7]. Thus, issues remain about the authentic role of mobile health. Most importantly, none of the studies specifically enrolled diabetic patients who had undergone CABG which are regarded as high-risk populations and usually manage multiple conditions, requiring several lifestyle and treatment recommendations [1].

Given the public health significance of poor diabetes management, high-risk profile in patients with concomitant diabetes after CABG and problems with previous studies, we design and conduct the glycaemic control using miniprogram-based intervention in patients with diabetes undergoing coronary artery bypass to promote self-management (GUIDE ME) study (NCT04192409).

The primary objective of this study is to evaluate the efficacy and feasibility of mobile-based coaching intervention, based on BCTs, in improving risk-factor control and secondary prevention in this high-risk group of patients.

## Methods

### Study overview

The ongoing GUIDEME study is a multi-centre randomized controlled trial of an automated mobile phone miniprogram-based intervention with 6 months of follow-up. We hypothesize that education and medication reminders for patients can help reduction of A1c over 6 months. This RCT is registered at http://www.clinicaltrials.gov (NCT04192409). The central ethics committee of Fuwai Hospital approved the study (No.2019–1151, June 2019). The recruitment and the last follow-up are expected to finish in June 2022 and December 2022, respectively. All participants provided written informed consent at the initial trial visit.

### Patient and public involvement

No patient was involved.

### Inclusion and exclusion criteria

Patients must meet all of the following criteria to be recruited for the study: (1) type 2 diabetes diagnosed by a physician prior to study enrollment; (2) documented coronary artery disease and isolated coronary artery bypass is recommended and performed; (3) access to a mobile phone with WeChat and ability to operate on the miniprogram. The participants are eligible if they are ≥ 18. Patients are excluded if they could not read the materials, had cognitive or communication disorders, and are unable to provide informed consent or die before discharge.

### Patient recruitment

We identify patients in the surgical ward who have been hospitalized with CHD and DM and who have undergone coronary artery bypass. The diagnoses of CHD and DM and the indication for surgical revascularization are adjudicated centrally, based on reviews of the patients’ medical charts. Once identified, study researchers explained the study face-to-face and that they may be eligible to participate. A ‘screening log’ of basic demographic information and reasons for not participating in patients deemed ineligible or declined to participate has been recorded. Individual patient signature informed consent is obtained by the China National for Cardiovascular Disease independent of clinical office involvement.

Based on previous experience [8], we estimate that surgical practice would provide an average of 1300 patients with CHD and DM undergoing CABG in Beijing Fuwai Hospital annually and 100 patients in Qingdao Fuwai Hospital.

### Allocation sequence generation

Participants are randomly allocated to enter the intervention or the control arm in a 1:1 ratio using a computerized randomization system using minimization. In order to achieve a balance of participants’ characteristics in both arms, a stratified randomization approach is employed, based on age, gender, education degree, acute myocardial infarction history and medical insurance type.

#### Allocation concealment mechanism

A computerized randomization system using the minimization method is developed to implement the allocation sequence.

#### Allocation implementation

A computerized randomization system using the minimization method will generate the allocation sequence. This allocation sequence will automatically assign participants into the intervention group or the control group. After this assignment, researchers will enrol participants and accomplish the assignment of participants to interventions.

#### Blinding

Statisticians and clinic staff are blinded to treatment allocation.

### Trial intervention

**Fig. 1:**
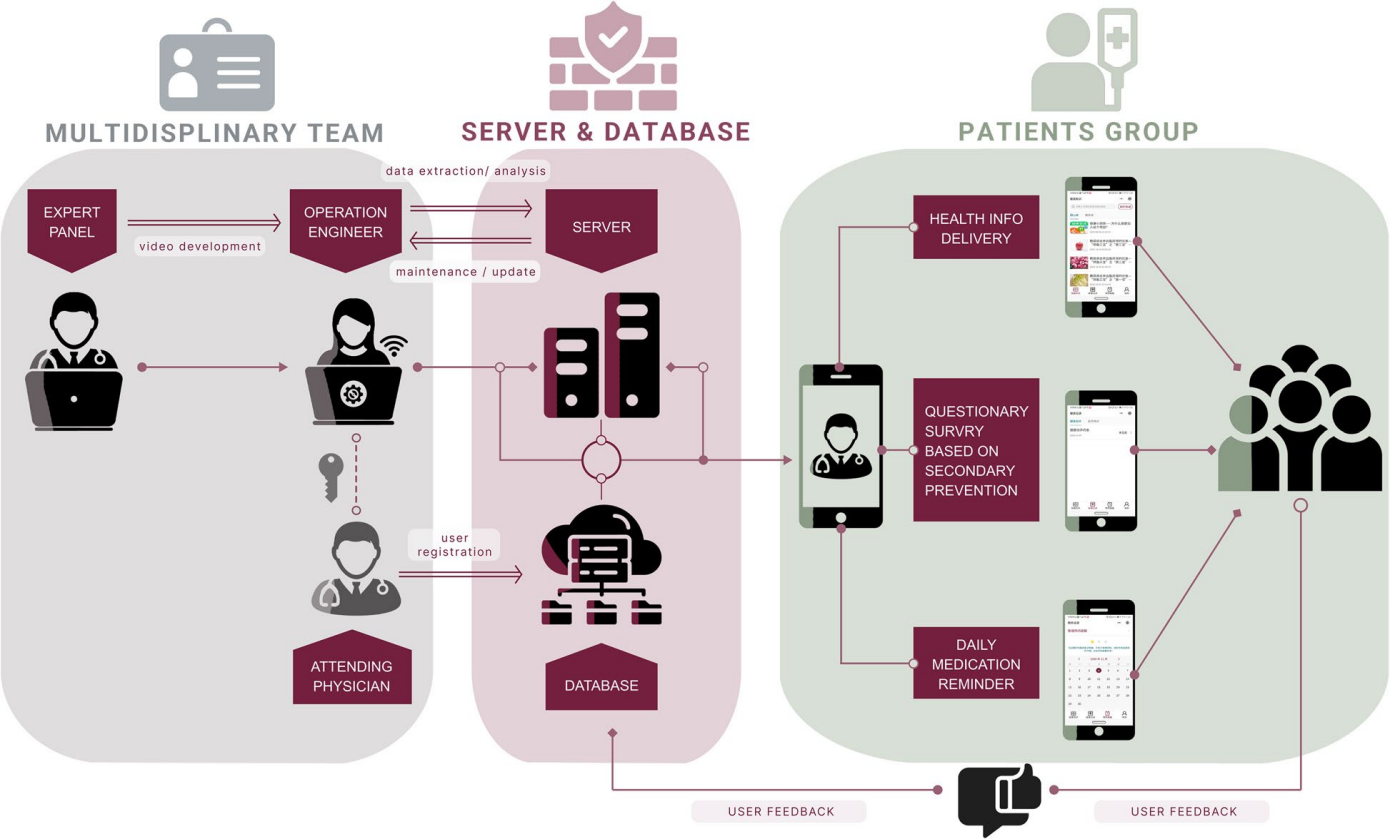
Schematic to show patient mobile connection, patient/physician web portals and servers

**Fig. 2:**
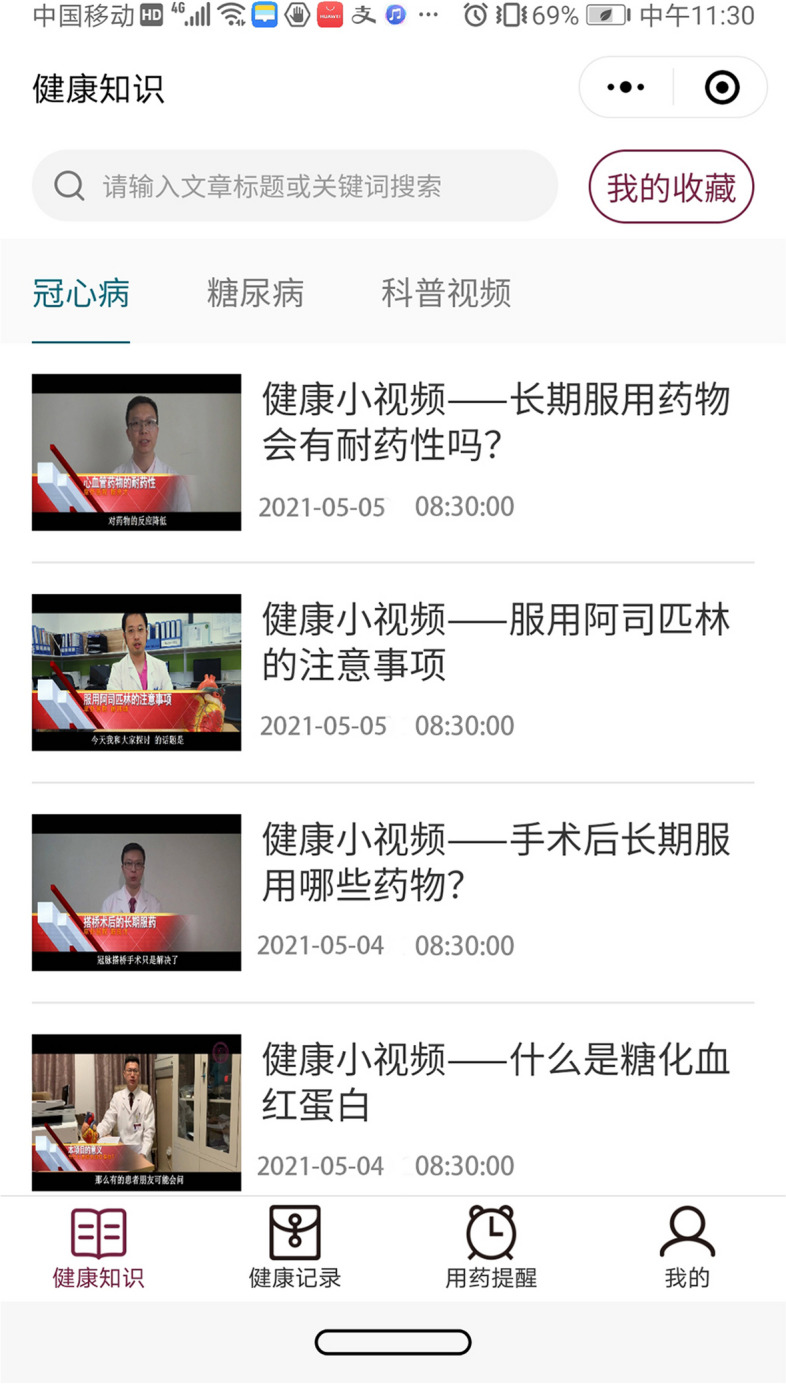
Functional modules of coaching and reminder on the WeChat terminal

The interventions are education and reminder systems for patients with CHD and DM, using mobile phones to allow transmission (Fig. 1). Participants in the intervention groups receive video (including lines) about CHD and diabetes risk modification education for 6 months as well as the standard treatment. The control group receive standard treatment. The standard treatment included (1) short education lesson regarding the secondary prevention recommendations after CABG during hospitalizations. Antiplatelet therapy, antithrombotic therapy, lipid management, β-blocker therapy, hypertension management, and diabetes mellitus management are explained including what-, why- and how-. The recommendations are consistent with the 2015 AHA scientific statement—secondary prevention after coronary artery bypass graft surgery [1]. (2) A brochure. In this brochure, preoperative, postoperative and discharge education notifications are documented. With regard to discharge education notification, diet, physical activity, wound care, follow-up, and medication advice are documented. All patients in the intervention groups receive the study treatment mobile phone mini-program software which is anchored in WeChat (Fig. 2). A training session is held by the research staff on enrollment that participants in the intervention group are registered in the system so that they have access to the web-based individual patient portal and they are capable of receiving and reading system-driven coaching video material. In addition, participants in the intervention receive medication regimen reminders. Prior to commencement, the system is tested to confirm functioning effectively. Researchers at the China National for Cardiovascular Disease could monitor the visiting frequency of the system. Participants are also informed that they could withdraw from the study by sending text messages to the research staff. Outbound patient phone calls by the research staff are discouraged.

### Intervention development

A cluster of 180 videos are developed by a multidisciplinary team of cardiac surgeons, cardiologists, endocrinologists, psychologists, nurses and public health researchers using a two-phase systemic and iterative approach. Coaching videos cover a range of secondary prevention after coronary artery bypass graft surgery in addition to diabetes self-management topics based on current guidelines [1, 9–13] including (1) general education on CHD and DM, (2) postoperative antiplatelet agents; (3) lipid-lowering therapy; (4) b-blocker therapy; (5) glucose monitoring and control, (6) blood pressure control, (7) smoking cessation; (8) cardiac rehabilitation; and (9) lifestyle recommendations such as weight loss, physical activity, diet. Other self-care behaviours including monitoring, self-management problem solving, reducing risks and healthy coping are considered as essential bahaviours for improving diabetes mellitus self-management and are incorporated into video development accordingly [14, 15]. All videos are in Chinese and are less than 2 min. The captions of the videos are affiliated and are also developed into text.

### Phase I: developing videos

Lines of the coaching videos are originally drafted in Chinese based on current guidelines. BCTs are employed to develop short videos including goal setting, providing information on consequences of behaviour, self-monitoring, barrier identification and social support [16, 17]. Table 1 illustrates the BCTs used in the lines of short videos. Videos were developed in form of lectures or dialogues in order to make them more acceptable to patients and compatible with Chinese beliefs and values [18]. Lectures begin with a question regarding to the subject and are followed by the response. Videos of dialogue are made in the setting of real-world examples instead of abstract theories [19]. These videos are sent to experts in BCTs and subsequently reviewed, criticized and revised within the research team.

**Table 1:** Behaviour change technique used in video development

Behaviour change technique	Content/explanation	Examples of lines of video in English
Provide information about behaviour-health risk	General information about behavioural risk	Aspirin should be continued indefinitely to reduce graft occlusion and adverse cardiac events. In order to convey the most benefit of CABG to you, taking medications regularly
Provide instruction	Tell the person how to perform a behaviour and/or preparatory behaviours	Smoking cessation is critical. Electronic cigarettes (e-cigarettes) have not been demonstrated to improve cessation rates and important concerns have been raised about their potential for adverse health effects. However, nicotine replacement therapy, such as bupropion and varenicline as adjuncts to smoking cessation for stable CABG patients after discharge is reasonable. And you can try this method!
Prompt barrier identification	Identify barriers to perform the behaviour and plan ways of overcoming them	b-Blockers are often associated with side effects such as weight gain, fatigue, and sexual dysfunction. You may feel upset about this and are reluctant to take it regularly. But if you have hypertention, a history of myocardial infarction or a history of heart failure, keep taking it regularly as this is good for your body. We will remind you to take this medication through miniprogram. And you can also set a repeating alarm on your cellphone
Prompt self-monitoring of behaviour	The person is asked to keep a record of the specified behaviour	Meals, exercise, cold and diarrhoea will make your blood sugar levels fluctuate. Thus, you should perform self-monitoring of blood glucose (SMBG) prior to meals and snacks, at bedtime, occasionally postprandially, prior to exercise and prior to critical tasks such as driving. You may as well upload this data through the mini-program. In addition, perform the A1C test 3 months and 6 months after surgery even if you have stable glycaemic control. Striving to achieve an HbA_1c_ is a reasonable goal for most patients after CABG
Set graded tasks	Set easy tasks, and increase the difficulty until target behaviour is reached	Have you ever felt hard to take medicine regularly? All things are difficult before they are easy. However, our mini-program system will help you. You can enter your medication and the system will automatically generate the ratings according to your medication adherence performance. More stars you get, the more benefits you will gain. Move on to get the most stars and it will make a great difference in the future!
Plan social support or social change	Prompt consideration of how others could change their behaviour to offer the person help or (instrumental) social support	Symptoms of hypoglycaemia include, but are not limited to shakiness, irritability, confusion, tachycardia, and hunger. Tell your friends and family when you have the symptoms above so that they give you the glucose (15–20 g). Fifteen minutes after treatment, if SMBG shows continued hypoglycaemia, the treatment should be repeated. Once SMBG returns to normal, the individual should consume a meal or snack. The family members should know where the glucagon is and how to administer it in case of blood glucose < 54 mg/dL
Stress management	May involve a variety of specific techniques (eg. Progressive relaxation) that do not target the behaviour but seek to reduce anxiety and stress	Relaxation is something we need to learn and practice. Listening to music, reading or talking to friends and family can ease stress
Motivational interviewing	Prompt the person to provide self-motivating statements and evaluations of their own behaviours to minimize resistance to change	Does your A1C decrease in value 3 months after CABG? If so, it is something worth celebrating. We are sure that you have put a lot of effort into taking medications and lifestyle modification. Keep up the good work and you can make a difference. If A1C remains less than 7%, it is supposed that you achieve the target of glycaemic control. However, less stringent A1C goals (such as < 8%) may be appropriate according to recent guidelines. Thus, glycaemic target must be individualized to the needs of each patient and his or her disease factor

### Phase II: expert review

The 180 videos are reviewed with a different focus by an expert panel consisting of clinicians and academics in the field of cardiology, endocrinology, epidemiology, psychology and linguistics. First, the clarity, accuracy and feasibility of each video is checked and verified. Second, linguists review all videos and focus on the acceptability and readability of the video education material. Issues regarding videos are addressed, and the corresponding education videos are shot again in order to achieve the desired target which the expert panel had proposed.

### Frequency and timing of education video delivery

One hundred eighty coaching videos in total are pushed forward to the participants in the intervention group across a 6-month timeframe. The videos are sent to the participant portal every 2 days in the morning. The theme of the coaching video covers a range of recommendations from the recent guidelines. Participants in the intervention group will receive the updated text message and log into the WeChat-based miniprogram to read the updated educational video material. Initially, only one education video is available. To evaluate the engagement in the miniprogram system, participants are required to finish the question at the end of every video, “Do you think that the information is easy to understand? Do you think that information is useful?”. The logbook data are analyzed at the completion of the study intervention. Through the 6-month follow-up, research staff call participants if they do not finish the questions for two consecutive weeks and only one call is made per patient during the intervention period, so as not to confound the intervention. Patient communication is delivered by automated feedback on the mobile phone WeChat-affiliated miniprogram and messaging through the message centre in the patient web portal.

### Data collection and management

Patient characteristics are collected from self-reported interviews with trained research staff and medical chart reviews including basic information, baseline clinical variables, biochemistry information, coronary angiography, echocardiographic outcomes, diabetes-related variables, surgical details, postoperative complication and socioeconomic status (Supplementary materials).

Additional assessments of baseline medication adherence, health status (EuroQol five-dimensional questionnaire: EQ-5D), are also conducted in person [20]. Biochemistry tests will be analysed at the central laboratory. The process of recruitment is monitored and data collection is checked by trained staff from China National for Cardiovascular Disease to improve the quality control.

A specialized software platform is established by the Information Technology team for use in sending coaching video materials to participants and also recording responses. In addition, project progress can be supervised and 24-h management support is provided through this web-based platform. Predesigned onscreen case report form is entered by two staff members independently, and data are then securely transmitted to the central server through automatic electronic transfer. Continuous checks are run to ensure that data being entered are complete and meet predefined data formats and ranges to ensure the reliability and validity of the data.

The database is regularly backed up and password protected so that only a limited number of approved staff members can have access to the data. Data confidentiality policies of NCCD on data collection, storage and analysis have been strictly imposed to ensure the confidentiality.

### Outcomes

The primary outcome is the change in glycaemic HbA_1c_ by the central blood sample. Secondary outcomes include blood pressure, blood glucose, low-density lipoprotein, a change in the proportion of patients achieving HbA_1c_ < 7% of patients, graft patency, major adverse cerebrovascular and cardiovascular events (MACCEs), change in medication adherence, mini-program behaviour adherence, changes in antihyperglycaemic medications during the intervention and health status (EQ-5D). MACCEs include death, non-fatal myocardial infarction, stroke and any repeated revascularization and cardiac rehospitalizations. HbA_1c_ is determined using a high-performance liquid chromatography technique with ADAMS A_1c_HA-8180 (ARKRAY, Japan). Graft outcomes are assessed by multislice computed tomographic angiography (MSCTA) or catheter coronary angiogram (CAB) at 6 months after coronary artery bypass grafting. The examinations are conducted according to standardized radiology and cardiology procedures. Digital images from a ≥ 64-slice CT scanner are analyzed with software (Intellispace portal Version 6.0, Phillips Healthcare). Members of the independent Image Data Review Centre reviewed the images and adjudicated the patency of the grafts blinded to treatment assignments.

Graft patency is defined according to FitzGibbon criteria [21]. In our trial, graft patency is defined as FitzGibbon grade A [22, 23]. Quality of life is measured using the short version of EQ-5D.

The institutional follow-up protocol requires that patients who are discharged alive visit our outpatient clinic 3 and 6 months after coronary artery bypass graft. If adverse events are reported, the patient medical records in the outpatient clinic were checked cautiously. When the patients visited another hospital, they are asked to send copies of their medical records by mail. If the patient dies at home without any evidentiary material, a structured summary of death conversation with family members would be reported. All information is sent to NCCD for central adjudication according to prespecified criteria by trained clinicians.

#### Access to data

The data and statistical code are not available to be shared at this time as no datasets are analysed during the current study. The final dataset can be available at the end of this study after contact with the corresponding author.

#### Ancillary and post-trial care

In our study, there is no provision for ancillary and post-trial care. The intervention group participants will not receive any reminder nor coaching video after the study.

### Statistical analysis

All analyses will be conducted according to the intention-to-treat principle. Baseline patient characteristics are represented as the means with standard deviations (SDs) for continuous variables and proportions for categorical variables. The primary analysis will use an analysis of covariance. For categorical secondary outcomes, log-binomial regression is used to compare groups and calculate the relative risk of outcomes at 6 months (ie, proportions of patients achieving HbA_1c_ < 7.0%). A generalized estimating equation model including terms for treatment is used to estimate between-group differences in graft patency and 95% confidence intervals (CIs). For the (time-to-event) secondary outcomes, hazard ratios (HRs) and corresponding 95% CIs are determined with Cox proportional hazards regression analysis. Kaplan–Meier curves are used to depict the occurrence of secondary outcomes over time. Follow-up of event-free patients with incomplete follow-up will be censored at the last clinical contact. Additionally, we performed prespecified subgroup analyses of outcomes by age (< 60 and ≥ 60 years), sex (male and female), area (urban and rural), education (≤ 12 and > 12 years) and tertile of baseline HbA_1c_. Subgroup results are presented as mean differences with 95% CIs. All tests of significance are 2-tailed, with an a of 0.05.

We estimate that a sample size of 820 would provide 80% power at the 5% significance level to detect a 0.3% absolute difference in HbA_1c_ change in the intervention group at 6 months, compared with the control group, assuming a mean HbA_1c_ level of 7.0% at baseline (SD 1.4%) based on data from studies involving similar populations [24], using PASS, version 11.0 (NCSS, Kaysville, UT), for sample size calculation. This sample size allows for a 20% loss to follow-up during the study period. We used the SPIRIT checklist when writing our report [25].

#### Dissemination policy

The trial results will be disseminated via publications. There is no publication restriction.

## Discussion

The GUIDEME Study aims to assess the efficacy of an innovative intervention for improving secondary prevention by using a mobile-based video-coaching miniprogram among patients with diabetes and CHD after coronary artery bypass in China. To the best of our knowledge, this is the first to investigate the efficacy of video coaching and medication reminder system to improve self-management in a high-risk group of patients with diabetes and CHD undergoing coronary artery bypass and has the potential to be applied in resource-limited settings across diverse populations.

The GUIDEME study has several strengths. Up till now, there is a paucity of large RCTs of mobile diabetes mellitus management. With regard to smaller studies, the type of mobile technologies used for diabetes mellitus self-management research interventions include mobile platforms with diabetes mellitus-specific software apps or short message service [6]. Meta-analysis showed that mobile phone interventions significantly reduced HbA_1c_ by a mean of 0.5% over a median follow-up of 6 months [26]. However, most clinical trials examined change in HbA_1c_ during a 3-month intervention and it may be inaccurate to assume that a significant change in HbA_1c_ in the intervention group is attributable to technology instead of other nonspecific benefits of participants considering a report from a 2011 survey that 26% of downloaded health apps are used only once and 74% are abandoned by the 10^th^ use [27]. In view of the shortcomings of software apps or short message services above, our study employs video coaching approach based on the WeChat miniprogram. WeChat is the most popular social media platform in mainland China, with over 1 billion active users [28]. Patients place more value on health service delivery and intervention program using WeChat has been effectively applied in a range of clinical settings [29–32]. Considering the fact that our patient population are more of older adults and the majority of adults prefer learning by following directive rules and guidelines [33], patient education and management in the form of video is prioritized instead of text message or software apps. Coaching videos are more acceptable and understandable than short messages. This has the potential to promote secondary prevention adherence.

Our study is further distinguished by the management of multiple risk factors. Prior studies evaluated the effectiveness of mobile computing and communication technologies among patients with one risk factor. Few studies focus on high-risk populations. In addition, the efficacy of mobile technology in the management of multiple risk factors has not been fully explored, which is a reality for many patients. One such group of patients pertains to diabetic patients undergoing coronary artery bypass who carry a high-risk profile. It is emphasized that such groups of patients require multifaceted strategies [34]. In addition, patient-centred cognitive behavioural strategies are recommended to help patients achieve lifestyle changes and practice self-management [34]. Thus, targeting multiple risk factors using mobile technology hold the promise for achieving this purpose. More importantly, a lack of evidence exists pertaining to the effectiveness of mobile intervention in high-risk of patients with concomitant diabetes and coronary artery disease requiring surgical revascularization.

Furthermore, the content of the coaching video in our study is theory-driven and culturally sensitive. It evaluates a coaching system using mobile phones to deliver treatment recommendations and behaviour support based on evidence-based guidelines. Two systematic reviews concluded that interventions were more likely to be successful if they selected and combined theory-based behaviour change strategies [35, 36]. However, very few studies specified a theoretical rationale on this specific population. The GUIDEME study is further distinguished by the large sample size and multi-centre research. Our study enrols patients from a range of geographically diverse countries which will reflect the real-world practice across China.

Several limitations need to be acknowledged. First, patients with vision or touch disability or those who have no access to mobile technologies are excluded from our study which leads to selection bias. However, our study aims to assess the efficacy of mobile technology. When the efficacy is justified, measures will be taken to meet the needs of the specific population groups above. Second, behavioural factors are not included in the study that may influence the initial engagement and ongoing use of mobile technology and its associated impact on outcomes. And medication adherence is measured by self-report which carries the possibility of recall bias and social desirability bias. However, we believe that any such factors would be balanced across the treatment and control groups.

The GUIDEME study has paramount public health implications. Patients with diabetes and coronary artery disease requiring surgical revascularization are at high risk for mortality and major vascular events. Provider coaching and reminders are associated with improvement in adherence to guidelines and with clinically significant improvements in patient outcomes [37]. The widespread distribution of mobile phones, across China, combined with their unique ability to process and communicate data in real-time, make them an ideal platform to create simple and effective diabetes management programs. The GUIDEME study may serve as important models for evidence-based public health interventions and could convey benefits to a diverse population in China.

## Conclusion

The ongoing GUIDEME study is a multicentre, randomized controlled trial and will testify to the efficacy of mobile coaching and reminder intervention to improve secondary prevention in patients with diabetes and coronary artery disease requiring surgical revascularization in China. If successful, such mobile intervention could be used and scaled up to improve care for this high-risk group of patients.

## Trial status

Protocol version number 1. Date: October 5 of 2021. The recruitment began in January 2020. The study ceased in February 2020 due to COVID-19 until September 2020. The recruitment will be completed approximately in June 2022.

### Supplementary Information

**Additional file 1: Supplementary materials.** Data collection.

**Additional file 2. **SPIRIT checklist.

## Data Availability

The data and statistical code are not available to be shared at this time as no datasets are analysed during the current study.

## Abbreviations

CABG: Coronary artery bypass grafting
CHD: Coronary artery disease
DM: Diabetes mellitus
LIMCs: Low- and middle-income countries
HbA_1c_: Glycaemic haemoglobin
