# Supplementary material for: Glycaemic control using mobile-based intervention in patients with diabetes undergoing coronary artery bypass—study protocol for a randomized controlled trial
Source: Trials. 2023 Sep 13;24:585. doi: 10.1186/s13063-023-07580-x (PMC10498600; doi:10.1186/s13063-023-07580-x)
Supplement: Supplementary file 1 — Additional file 1: Supplementary materials. Data collection. [file 13063_2023_7580_MOESM1_ESM.docx]

Supplementary materials

*Data collection*

| Column | Variables |
| --- | --- |
| Basic information | Identity number |
|  | Age |
|  | Women |
|  | Patient number |
|  | Address |
|  | Educational attainment |
|  | Medical insurance |
| Clinical information | Body mass index |
|  | Resting blood pressure |
|  | Canadian Cardiovascular Society Angina Classification |
|  | New York Heart Association classification |
|  | Prior atrial fibrillation |
|  | Prior stroke |
|  | Previous myocardial infarction |
|  | Preoperative creatinine |
|  | Previous open heart surgery |
|  | Peripheral arterial disease |
|  | Hypertension |
|  | Hyperlipidemia |
|  | Chronic obstructive pulmonary disease |
|  | Smoking |
| Echocardiography | Ejection fraction (%) |
|  | Left ventricular end-diastolic dimension (LVEDD) |
|  | Mitral valve regurgitation (mild, moderate, moderate to severe, severe) |
| Medications | ACE inhibitor/ARB |
|  | Aspirin |
|  | β-Blocker |
|  | Statin |
|  | Clopidogrel |
|  | Insulin |
|  | Oral antidiabetic medication |
| Diabetic complication | Retinopathy |
|  | Diabetic nephropathy |
|  | Diabetic foot |
| Biochemistry data | Hemoglobin A1C |
|  | Diabetic duration (years) |
|  | Mean cholesterol, mmol/L |
|  | Mean LDL, mmol/L |
| Coronary angiography | No. of diseased vessels (0, 1, 2, 3) |
|  | Left main disease |
| Health status | EQ-5D |
| Intraoperative data | On-pump |
|  | No. of grafts bypassed |
|  | Left internal thoracic artery or right internal thoracic surgery |
|  | Left internal thoracic artery and right internal thoracic surgery |
|  | Radial artery |
|  | Transit time flow |
| Perioperative data | Length of ICU stay |
|  | Length of hospital duration |
|  | Blood transfusion |
|  | Re-operation for bleeding |
|  | Prolonged ventilation (>24h) |
|  | Stroke |
|  | Myocardial infarction |
|  | Atrial fibrillation |
|  | Deaths |

EQ-5D, EuroQol 5-Dimensional Questionaire; ICU, intensive care unit; LDL, low-density lipoprotein cholesterol.
